# Ambient air pollution and daily mortality in ten cities of India: a causal modelling study

**DOI:** 10.1016/S2542-5196(24)00114-1

**Published:** 2024-07

**Authors:** Jeroen de Bont, Bhargav Krishna, Massimo Stafoggia, Tirthankar Banerjee, Hem Dholakia, Amit Garg, Vijendra Ingole, Suganthi Jaganathan, Itai Kloog, Kevin Lane, Rajesh Kumar Mall, Siddhartha Mandal, Amruta Nori-Sarma, Dorairaj Prabhakaran, Ajit Rajiva, Abhiyant Suresh Tiwari, Yaguang Wei, Gregory A Wellenius, Joel Schwartz, Poornima Prabhakaran, Petter Ljungman

**Affiliations:** Institute of Environmental Medicine, Karolinska Institutet, Stockholm, Sweden (J de Bont PhD, S Jaganathan MPH, P Ljungman PhD); Sustainable Futures Collaborative, Delhi, India (B Krishna DrPH); Department of Epidemiology, Lazio Region Health Service /ASL Roma 1, Rome, Italy (M Stafoggia PhD); Institute of Environment and Sustainable Development, Banaras Hindu University, Varanasi, India (T Banerjee PhD); Smart Prosperity Institute, University of Ottawa, ON, Canada (H Dholakia PhD); Public Systems Group, National Investment & Infrastructure Fund Chair in Environment, Social & Corporate Governance, Indian Institute of Management, Ahmedabad, India (A Garg PhD); Environmental, Climate, and Urban Health Division, Vital Strategies, New York, NY, USA (V Ingole PhD); Office for National Statistics, Newport, Wales, UK (V Ingole); Centre for Chronic Disease Control, New Delhi, India (S Jaganathan, S Mandal PhD, Prof D Prabhakaran DM, A Rajiva MESc, P Prabhakaran PhD); Public Health Foundation of India, New Delhi, India (P Prabhakaran); Ashoka University, Sonipat, India (S Jaganathan, S Mandal, A Rajiva, P Prabhakaran); Ben-Gurion University of the Negev, Beer-Sheva, Israel (Prof I Kloog PhD, A Rajiva); Department of Environmental Medicine and Climate Science, Icahn School of Medicine at Mount Sinai, New York, NY, USA (Prof I Kloog, Y Wei PhD); Department of Environmental Health, Boston University School of Public Health, Boston, MA, USA; (Prof A N-Sarma PhD, Prof G A Wellenius PhD, K Lane PhD); DST-Mahamana Center of Excellence in Climate Change Research, Institute of Environment and Sustainable Development, Banaras Hindu University, Varanasi, India (R K Mall PhD); NRDC India Private Limited, New Delhi, India (A S Tiwari MPH); Department of Environmental Health, Harvard T.H. Chan School of Public Health, Boston, MA, USA (Y Wei, Prof J Schwartz PhD); Department of Cardiology, Danderyd Hospital, Stockholm, Sweden (P Ljungman)

## Abstract

**Background:**

The evidence for acute effects of air pollution on mortality in India is scarce, despite the extreme concentrations of air pollution observed. This is the first multi-city study in India that examines the association between short-term exposure to PM_2·5_ and daily mortality using causal methods that highlight the importance of locally generated air pollution.

**Methods:**

We applied a time-series analysis to ten cities in India between 2008 and 2019. We assessed city-wide daily PM_2·5_ concentrations using a novel hybrid nationwide spatiotemporal model and estimated city-specific effects of PM_2·5_ using a generalised additive Poisson regression model. City-specific results were then meta-analysed. We applied an instrumental variable causal approach (including planetary boundary layer height, wind speed, and atmospheric pressure) to evaluate the causal effect of locally generated air pollution on mortality. We obtained an integrated exposure–response curve through a multivariate meta-regression of the city-specific exposure–response curve and calculated the fraction of deaths attributable to air pollution concentrations exceeding the current WHO 24 h ambient PM_2·5_ guideline of 15 μg/m^3^. To explore the shape of the exposure–response curve at lower exposures, we further limited the analyses to days with concentrations lower than the current Indian standard (60 μg/m^3^).

**Findings:**

We observed that a 10 μg/m^3^ increase in 2-day moving average of PM_2·5_ was associated with 1·4% (95% CI 0·7–2·2) higher daily mortality. In our causal instrumental variable analyses representing the effect of locally generated air pollution, we observed a stronger association with daily mortality (3·6% [2·1–5·0]) than our overall estimate. Our integrated exposure–response curve suggested steeper slopes at lower levels of exposure and an attenuation of the slope at high exposure levels. We observed two times higher risk of death per 10 μg/m^3^ increase when restricting our analyses to observations below the Indian air quality standard (2·7% [1·7–3·6]). Using the integrated exposure–response curve, we observed that 7·2% (4·2%–10·1%) of all daily deaths were attributed to PM_2·5_ concentrations higher than the WHO guidelines.

**Interpretation:**

Short-term PM_2·5_ exposure was associated with a high risk of death in India, even at concentrations well below the current Indian PM_2·5_ standard. These associations were stronger for locally generated air pollutants quantified through causal modelling methods than conventional time-series analysis, further supporting a plausible causal link.

**Funding:**

Swedish Research Council for Sustainable Development.

## Introduction

Exposure to air pollution is a global public health hazard, with a considerable body of evidence linking short-term and long-term exposures to a range of health outcomes, including all-cause and cause-specific mortality, respiratory and cardiovascular conditions, neurodevelopmental deficiencies, and adverse pregnancy and birth outcomes.^1–6^ Evidence of these health harms has led to sustained reductions in air pollution exposures globally, yet many low-income and-middle income countries, including India, continue to experience high concentrations of air pollution.

Air pollution levels in many parts of India routinely exceed the WHO guidelines for safe exposure (24 h ambient PM_2·5_ standard of 15 μg/m^3^ not to be exceeded more than three to four times per year), and even exceed India’s own less stringent ambient air quality standards for 24 h ambient exposure (60 μg/m^3^).^7,8^ Annual average exposure to PM_2·5_ in the nation’s capital Delhi exceeded 100 μg/m^3^ in 2021 (WHO guideline value 5 μg/m^3^; Indian standard 40 μg/m^3^), with similar con centrations faced across much of the Indo-Gangetic Plain airshed.^9^ Meteorological factors and seasonal high combustion events, such as festivals or crop residue burning, often push short-term exposures to concentrations as high as 700–1000 μg/m^3^.^10^ These hyperlocal pollution episodes that trigger greater exposures, especially to ambient air pollution, can cause increased vulnerability and burden of disease. The 2019 subnational burden of disease study estimated that more than 10·4% of total deaths (approximately 980 000) and 6·7% of total disability-adjusted life years (approximately 31·1 million) are associated with exposure to ambient PM_2·5_.^10^ These estimates are treated with relative scepticism by policy makers because they are not based on studies from India. However, a growing body of local evidence has begun to fill the gaps in knowledge on both long-term and short-term exposures.^11–14^

Many studies elsewhere have evaluated the effect of short-term ambient air pollution on daily mortality. Although many of these studies are focused on specific geographical areas, some have conducted multi-city analyses in the USA, Latin America, Europe, China, and globally.^15–19^ To the best of our knowledge, there have been no multi-city studies conducted in India, and neither have any Indian cities featured in global multi-city, multi-country analyses. Previous studies on the effect of short-term PM_2·5_ exposures on daily mortality in India are scarce—they have focused only on one or two cities and have not investigated exposure–response curves across a broader range of exposures.^11,20^ Further, there are only a few studies that have evaluated the possible effect of locally generated air pollution on mortality through causal modelling techniques such as instrumental variable analysis.^21,22^ The instrumental variable approach relies on the selection of a variable (the instrument) that can cause a build-up of locally generated pollution but does not have other plausible links with daily changes in mortality, except through air pollution itself.^21,22^ In effect, the instrument allows local pollutants to vary independently in relation to both measured and unmeasured confounders, thus eliminating any effects that might influence the relationship between exposure and outcome. This approach allows us to provide causal estimates of the effect of changes in local air pollution levels.

Using a national spatiotemporal exposure model and daily mortality data from ten cities, we aimed to conduct the first multi-city analysis for India, including the use of causal modelling methods. The ultimate goal of our study was to provide a first national causal exposure–response function directly relevant to policy. Furthermore, the inclusion of cities with different exposure levels aimed to increase statistical power and capture a broader range of daily exposure to PM_2·5_.

## Methods

### Daily mortality

We obtained daily counts of all-cause mortality from the death registries of ten municipal corporations in India (Ahmedabad, Bangalore, Chennai, Delhi, Hyderabad, Kolkata, Mumbai, Pune, Shimla, and Varanasi), covering each of the five climate zone classifications (appendix p 2). The data covered the period from 2008 to 2019, with 3–7 years of data available for each city (appendix p 2). We acquired de-identified mortality records from each municipal corporation, and we cleaned and aggregated the data to compile daily deaths for use in our analyses. International Classification of Diseases codes were not available for most cities, leaving us unable to conduct analyses of cause-specific mortality. The city-specific populations varied from 170 000 in Shimla to approximately 16·8 million in Delhi.^23^

### Exposure assessment: daily ambient air pollution

We generated daily average PM_2·5_ concentrations at 1 km^2^ spatial resolution across India using a hybrid ensemble averaging approach from 2008 to 2020.^8^ Briefly, we collected ground monitoring-based observations of daily average PM_2·5_ and PM_10_ across 1056 locations and an extensive set of predictors encompassing satellite-based observations, meteorology, land-use patterns, emissions inventories, and reanalysis-based data. Using a cross-validation approach by leaving out 20% of the monitors, we trained four machine learning methods (deep learning, random forests, gradient boosting, and extreme gradient boosting) on the training data. The optimised models were implemented on the left-out validation data to obtain learner-specific predictions and combined using a Gaussian process regression to obtain the final predictions. The ensemble averaging was done to borrow strength across the different machine learning algorithms. We observed that certain algorithms performed better in specific areas and used a Gaussian process-based model (including elevation and land-use features) to combine the predictions from the four different algorithms into one final prediction for each grid-day combination. This method allowed us to obtain PM_2·5_ exposures in regions with no monitoring data across time. The daily ensemble averaged predictions had a cross-validated R^2^ of 86% and mean absolute error ranging between 14·4 μg/m^3^ and 25·4 μg/m^3^ across India. In this study, we estimated daily population weighted PM_2·5_ concentrations of all 1 km^2^ grid cells contained within the municipal boundaries of each of the ten cities included in the study throughout our study period. Population-weighted averages were used to provide a more accurate representation of the actual exposure experienced by the population.

### Analytical strategy

We applied a two-stage analysis approach to evaluate the effects of PM_2·5_ on daily mortality counts. In the first stage, we used quasi-Poisson generalised additive models (GAMs) to estimate city-specific associations. The models were adjusted for a penalised spline smooth function of calendar day with nine degrees of freedom (df) per year to account for underlying long-term and seasonal time trends, an indicator of day-of-week to account for weekly variations, and a natural spline function with four df for daily mean air temperature (lag 0–4). We used the 2-day moving average of current and previous day PM_2·5_ concentration (lag 0–1) to estimate the effect on daily mortality, in line with the current literature.^15,16^ We explored different lag patterns including single lags of same day (lag 0), previous day (lag 1), 2 days preceding level (lag 2), and 4-day moving average (lag 0–3). We modelled PM_2·5_ as a linear term, and expressed the effect estimates as percentage change in daily mortality, with 95% CIs per 10 μg/m^3^ increase in PM_2·5_ (lag 0–1). In the second stage, we applied a random-effects meta-analytical model to pool the city-specific estimates of associations of PM _2·5_ with mortality. We calculated *I*^2^ statistics and Cochran’s Q-test to evaluate the between-city heterogeneity.

### Effect of locally generated pollutants using instrumental variable analysis

We used an instrumental variable approach to estimate the causal effect of locally generated air pollution in India. A more comprehensive overview of this approach can be found elsewhere;^21,22^ briefly, the authors identified three instruments: planetary boundary layer height (PBLH), wind speed, and atmospheric pressure. PBLH is the elevation height at which vertical mixing of local emission occurs in the atmosphere. The mean PBLH varies day to day through dynamic interplay of various atmospheric processes. Wind speed affects horizontal transport of pollutants, with lower speeds increasing local influence and higher speeds promoting turbulent mixing and reduced concentrations. High atmospheric pressure typically induces weather conditions such as lower vertical temperature gradients, which impede both vertical and horizontal mixing of pollutants. Although each variable can individually capture distinct aspects of air pollution variation, the daily variability of each instrument is unlikely to be associated with daily deaths except through air pollution changes. Therefore, these three instruments serve as the most appropriate options for an instrumental variable in our study.^21,22^ If these variables are not predictors of mortality except through air pollution, then they should not be associated with any confounders. If these instruments produce variations in air pollution that are randomised with respect to measured and unmeasured confounders, and if that fraction of variation in air pollution is associated with daily mortality, the effect estimates should be causal.

We obtained daily mean levels of PBLH, wind speed, and atmospheric pressure from the European Centre for Medium-Range Weather Forecasts.^21,22^ Similar to PM_2·5_, we used the 2-day moving average of current and previous day PBLH, atmospheric pressure, and wind speed. We regressed our PM_2·5_ values (lag 0–1) on time trends, air temperature (lag 0–4), and day of the week, then extracted the residuals. To obtain a single final instrumental variable, the three instruments were combined to derive one single pollution-calibrated instrumental variable by applying a support vector regression (SVM) with a radial kernel to account for non-linear interaction between the predictors and the residuals of local pollution. We used the SVM function in the R package e1071. The obtained fitted values represent the remaining variation in PM_2·5_ that was explained by the three instrumental variables, and are independent of season, time trend, and temperature.^21,22^ Then, we used the instrument as our exposure in the quasi-Poisson regression in each city as specified previously. The effect estimates obtained from this model are on the same scale as PM_2·5_.

### Meta-analytic regression and attributable fraction

We assessed the shape of the exposure–response curve for each city using our main GAM. To account for possible non-linearity, we applied a quadratic B-spline with one single knot located at the 50th percentile of the city-specific air pollution distribution (2-day moving average of PM_2·5_). Then we applied a multivariate meta-regression of the city-specific predictions of the exposure–response curve to obtain an integrated exposure–response curve.^24^ As we observed a supralinear relationship, we used the integrated exposure–response curve to calculate the fraction of deaths attributable to air pollution concentrations exceeding the WHO 24 h ambient PM_2·5_ guideline of 15 μg/m^3^.^25^ To do so, for each day in each city, we used the overall integrated relative risk comparing each day’s air pollution with WHO guidelines to calculate the attributable deaths and attributable fraction, using a previously described method.^25^ Then, we obtained the total deaths attributable to PM_2·5_ above the WHO guidelines by summing all the daily attributable deaths series, and estimated the total attributable fraction by dividing the total number of attributable deaths by the total deaths. 95% CIs were derived through 1000 Monte Carlo simulations. Finally, we investigated if associations persisted at successively lower concentrations of air pollution (<250 μg/m^3^, <125 μg/m^3^, <100 μg/m^3^, <75 μg/m^3^, and <60 μg/m^3^, the last being the Indian standard of 24 h ambient PM_2·5_ concentration).

### Sensitivity analysis

To assess the robustness of our results we performed several sensitivity analyses. We applied different df (between six and ten df per year) to account for time trends, and we applied different adjustments for temperature (at lag 1 and 3 and using three and six df in the smoothing variables). Relative humidity is used as a confounder in previous studies, but these data were not available for all cities or all time periods.^9,23^ Thus, as a sensitivity analysis, we adjusted for relative humidity from meteorological stations for those cities when data were available (Ahmedabad, Bangalore, and Hyderabad). Finally, we estimated the integrated exposure–response curve and attributable fraction using different knot points for PM_2·5_, with equidistant knots (at 25th, 50th, and 75th percentiles) and at specific percentiles (10th, 50th, and 90th). We also estimated fractions of deaths attributable to air pollution concentrations exceeding the Indian 24 h ambient PM_2·5_ standard.

### Role of the funding source

The funder of the study had no role in study design, data collection, data analysis, data interpretation, or writing of the report.

## Results

**Figure 1:**
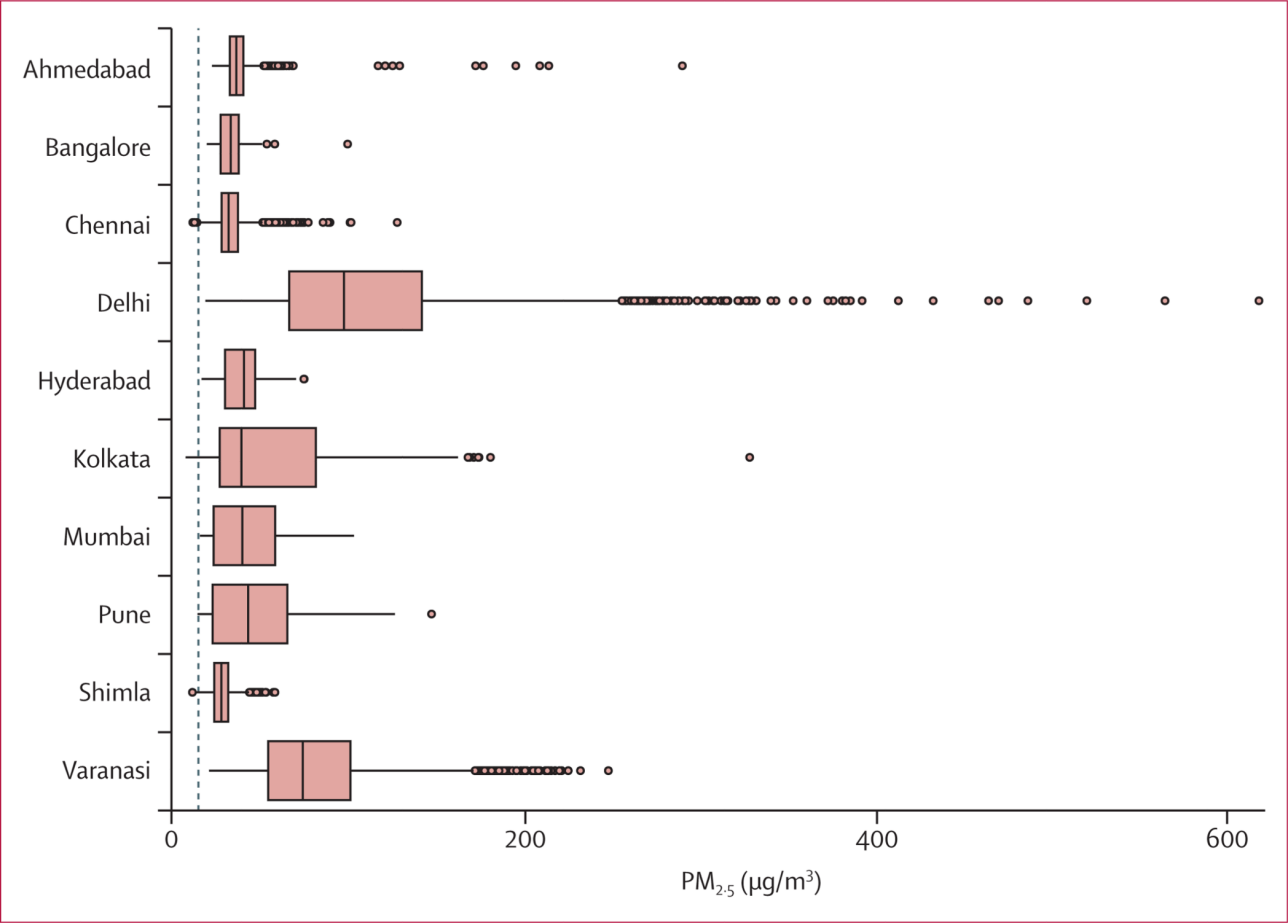
Daily PM _2·5_ concentrations across ten Indian cities (dashed line shows the WHO recommended air quality guidelines [24 h of 15 μg/m^3^]) Boxplot showing the median, IQR, minimum, maximum, and extreme values of PM_2·5_ concentrations. Extreme PM_2·5_ events, which significantly exceed the WHO guidelines, are particularly observed in cities such as Ahmedabad, Delhi, Kolkata, and Varanasi.

This time series analysis included more than 3·6 million deaths in India from 2008 to 2019 (appendix p 2). The long-term average of daily means of PM_2·5_ over this period ranged from 28·4 μg/m^3^ in Shimla to 113·0 μg/m^3^ in Delhi (figure 1). The maximum daily PM_2·5_ concentration was registered in Delhi at 617·6 μg/m^3^, and in 99·8% of all days across all cities (27 091 of 27 146 days) the daily PM_2·5_ concentrations exceeded the 2021 WHO recommended 24 h air quality guidelines of 15 μg/m^3^ (figure 1).

**Figure 2:**
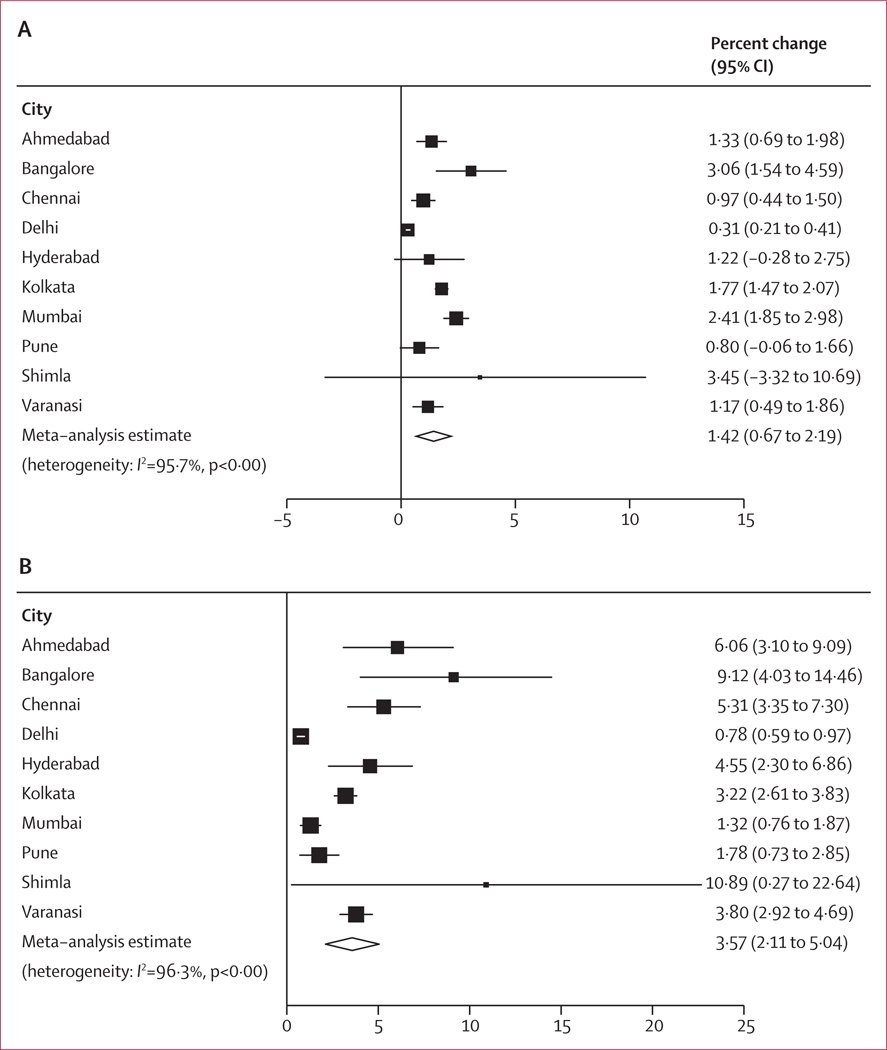
City-specific and pooled estimates using conventional time-series analyses (A) and instrumental variables causal analyses (B) of the association between short-term exposure to PM _2·5_ and daily mortality per 10μg /m^3^

From our main analyses, we observed a 1·42% (95% CI 0·67–2·19, *I*^2^ 95·7%) increase in daily mortality per a 10 μg/m^3^ increase of PM_2·5_ (lag 1; figure 2). The city-specific estimates showed large variations, ranging from 0·31% (0·21–0·41) in Delhi to 3·06% (1·54–4·59) in Bangalore. In our instrumental variable analysis, we observed an increase in daily mortality of 3·57% (2·11–5·04, 96·3%) per 10 μg/m^3^, which was higher than in the conventional time-series analyses (figure 2). The causal effects were especially strong in cities with lower concentrations of air pollution, such as Bangalore, Chennai, and Shimla.

Estimates are provided as percentage change in mortality and 95% CIs per 10 μg/m^3^ increase in PM_2·5_ (lag 1). Models were adjusted for a penalised spline smooth function of calendar day with nine df, an indicator of day-of-week, temperature (lag 4), and relative humidity (lag 4).

**Figure 3:**
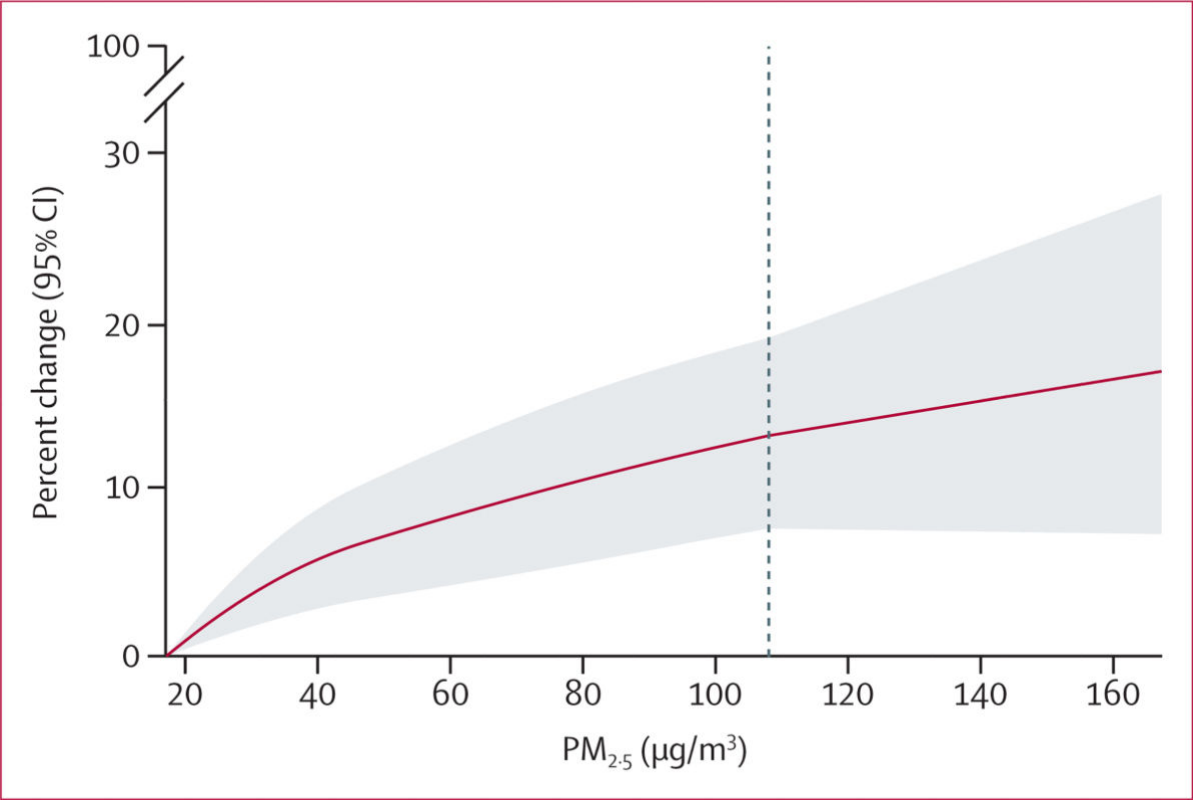
Integrated exposure–response curve (2-day moving average) between air pollution and mortality, with 95% CIs The figure represents the relative risk of air pollution against the minimum air pollution concentration (grey area) at which an effect was observed in our study (17·1 μg/m^3^). Ideally, this would be assessed at the WHO 24 h ambient PM _2·5_ guideline of 15 μg/m^3^, but this was not feasible as there were very few days where such concentrations were observed in our dataset. The dashed line shows the 99th percentile.

**Figure 4:**
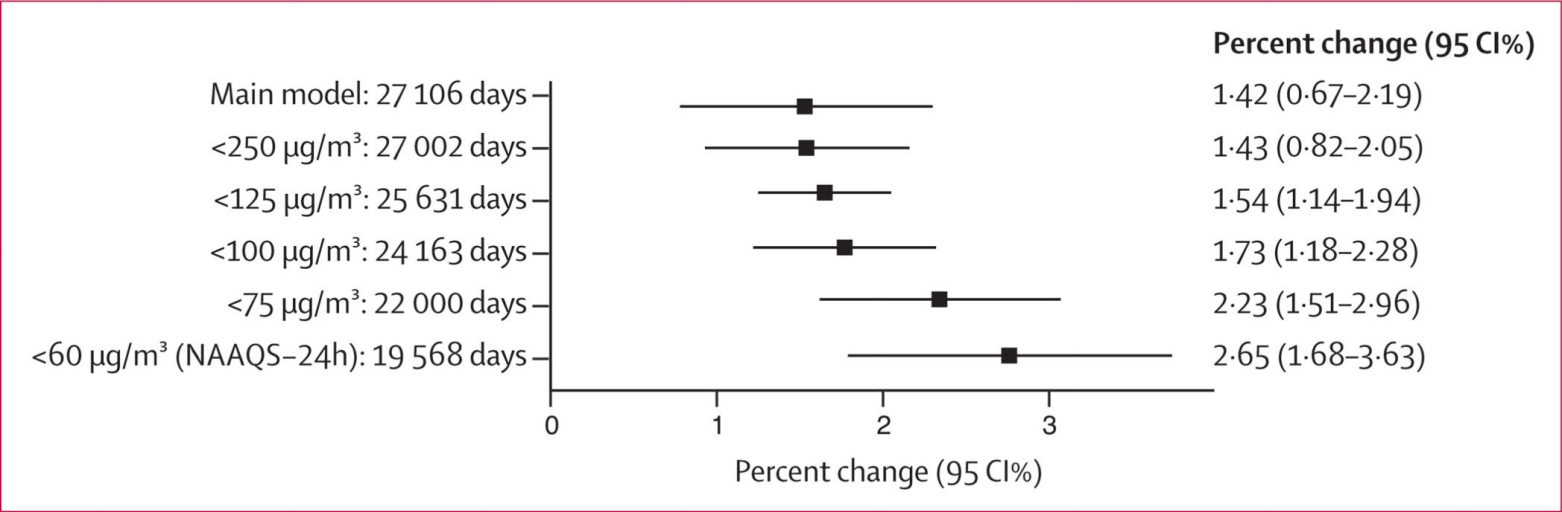
The effect of air pollution on daily mortality at lower thresholds of PM _2·5_ Days with daily PM_2·5_ concentrations above the selected thresholds were excluded. Models were adjusted for a penalised spline smooth function of calendar day with nine degrees of freedom, an indicator of day-of-week, temperature (lag 0–4) and humidity (lag 0–4). The Indian National Ambient Air Quality Standards recommend 24 h average PM_2·5_ concentrations to not exceed 60 μg/m^3^. The number of days at each threshold of PM_2·5_ per city are added in the appendix (p 4).

We observed a supralinear relationship in our integrated exposure–response curve, with steeper slopes at lower levels of exposure and an attenuation of the slope at higher levels of exposure (figure 3). We looked at the relative risk of air pollution against the minimum air pollution concentration at which an effect was observed in our study (17·1 μg/m^3^), as selecting the WHO 24 h ambient PM_2·5_ guideline of 15 μg/m^3^ was not feasible as there were not enough days in which such concentrations were observed in our dataset (figure 3). Using the estimated integrated exposure–response curve, we estimated that 7 ·2% (95 % CI 4·2–10·1) of all deaths were attributable to PM_2·5_ concentrations higher than the WHO recommended 15 μg/m^3^, corresponding to 33 627 (19 443–47 426) annual deaths across our ten cities (table). Delhi had the largest attributable fraction and highest attributable yearly deaths. The steeper slope at lower levels of exposure was supported when we restricted our analyses at different thresholds as we observed an increase in the effect estimates as we lowered the thresholds. When we restricted our analyses to days that observed PM_2·5_ concentrations below the recommended Indian guidelines (<60 μg/m^3^ recommended daily PM_2·5_ concentrations), we observed two times higher risk estimates compared with our main analyses without restriction (percent change [<60 μg/m^3^] of 2·65 [95%CI 1·68–3·63] per 10 μg/m^3^; figure 4).

Exploring different lag patterns, we observed similar associations for single lags of 0 and 1 days and lag 0–3 on daily mortality, but we observed a smaller effect on lag 0–2 days (appendix p 5). In the sensitivity analyses, we observed almost identical effect estimates adjusting for different df per year for time trend (six to ten df), and similar effect estimates were observed by adjusting for different degrees of smoothness for temperature (appendix p 6). The effect estimates of PM_2·5_ and mortality did not change after adjusting for relative humidity (appendix p 7). Finally, when using different knot points for PM_2·5_, we observed similar integrated exposure–response curves, but slightly higher attributable fractions and total attributable deaths (appendix p 8). Using the Indian standard, we observed lower deaths attributed to PM_2·5_ concentrations higher than 60 μg/m^3^ compared with the WHO guidelines (appendix p 3).

## Discussion

Our study analysed the association between PM_2·5_ exposure and approximately 3·6 million daily deaths in ten Indian cities between 2008 and 2019. As such, it is the first multi-city study to examine the association between short-term exposures to air pollution and daily mortality in India. We observed a clear association between daily PM_2·5_ exposure and increased risk of mortality. These associations were stronger when using causal modelling methods incorporating instrumental variables that isolated the effect of locally generated air pollution, indicating that previous studies probably underestimated the effect of short-term exposure to air pollution on daily mortality. Exposure–response curves generated as part of this study show the risk of mortality escalated rapidly at lower levels of exposure and tapered off at higher levels.

Overall, we found an increase of 1·42% (95% CI 0·67–2·19) in daily mortality associated with each 10 μg/m^3^ PM_2·5_ exposure. This effect estimate is higher than those reported by previous studies conducted in India^11,12,26^ and is higher than a recently published multi-city meta-analysis (499 cities) that reported a pooled estimate of 0·68% increase in daily mortality per 10 μg/m^3^ increase in PM_2·5_.^15^ When compared with regions that experience similar concentrations of PM_2·5_ exposure as India, our estimate remained higher, with a 272-city study in China reporting a 0·22% increase, and an 11-city east Asian study reporting a 0·38% increase in daily mortality per 10 μg/m^3^ increase in PM_2·5_.^16^ However, our effect estimate was lower than some country-specific effect estimates from Greece (2·54%), Japan (1·42%), and Spain (1·96%).^15^ Several factors could explain the stronger effects of PM_2·5_ observed in our study, including the differential composition and toxicity of PM_2·5_, varied age structures and susceptibility patterns, and climatological differences. We also found substantial heterogeneity in effect estimates across the cities studied, indicating the need for further research on local PM_2·5_ mortality associations, particularly since different cities have different pollutant source profiles.

Our integrated exposure–response curve showed a plateauing of risk at higher concentrations of PM_2·5_ exposure, similar to other city-specific studies from India and multi-city studies published elsewhere.^11,12,26^ For instance, the Chinese study of 272 cities had similar annual concentrations of PM_2·5_, but our exposure–response curve plateaus at higher concentrations of PM_2·5_.^16^ In addition, we observed stronger effects in lower polluted areas, such as Shimla and Bangalore, than higher polluted areas such as Delhi. This effect is probably related to the supralinear exposure–response curve, since Shimla and Bangalore had considerably lower concentrations. This sharp increase in risk at lower levels of exposure, which plateaus at higher levels, was reported by other studies in the region and studies in Europe.^12,14,25^

Analysis of the same relationship using an instrumental variable yielded a much higher effect estimate than the conventional time-series analysis. This difference could be due to several factors. First, the instrumental variable might be better at capturing the effect of locally generated air pollution because the instruments (planetary boundary layer height, wind speed, and atmospheric pressure) are directly related to higher contributions to ambient PM_2·5_ from local sources since they cannot be easily dispersed, and since when the boundary layer is low, transported pollution from elsewhere is generally not mixed downwards to the surface. It is likely that given the plurality of local sources observed in most Indian cities (including waste burning, local transport, and diesel generator sets), the air pollution generated from these sources might be more toxic than transported particles. However, this hypothesis requires further study. Second, it is possible that our model using the instrumental variable might capture the effect of other local air pollutants—such as NO_2_—and not just PM_2·5_. Since our model does not generate estimates of local NO_2_, we were unable to study the so-called cocktail effect^27^ of both pollutants, and we highlight the need for further study of this complex area.

As the first multi-city, time series analysis of short-term exposure to PM_2·5_ and daily mortality in India, our study has several strengths. First, the large dataset comprising approximately 3·6 million deaths provided us with more than adequate statistical power to estimate the observed effects. Second, we developed and used an innovative spatiotemporal exposure model to estimate PM_2·5_ concentrations. This model allowed us to move beyond the use of fixed site monitors and to generate population-weighted exposure metrics for each of the cities we studied. Third, through the use of instrumental variables, we have been able to generate causal estimates for the association between PM_2·5_ and mortality, providing deeper insight on the role of local sources of PM_2·5_ in this relationship.

Our study also had some limitations. First, although we were able to use our spatiotemporal exposure model to generate 1 km^2^ gridded predictions of PM_2·5_, the exposure metrics used in this study were daily city-level average PM_2·5_. This limitation is likely to have resulted in some non-differential misclassification of exposure, thereby lowering our effect estimates. Second, there is heterogeneity in the strength of death registration across the various states and cities in India, resulting in a pro portion of deaths being missed by the civil registration system each year. We expect that these deaths are probably missed at random in relation to daily variations in air pollution concentrations and unlikely to bias our effect estimates.^28,29^ Third, we were unable to obtain data for more cities and larger time periods, and on age, sex, and other individual-level effect modifiers, the analysis of which could have yielded information relevant to policy. For instance, analysis of effect modification of the PM_2·5_ mortality relationship in Delhi revealed a larger effect among elderly and male populations.^11^ As additional health data and contextual information become increasingly accessible in India, we anticipate that forthcomin g studies will have the opportunity to address these limitations. Finally, the minimum PM_2·5_ concentration observed across all cities in our study was 17·1 mg/m^3^, and this therefore served as the counterfactual for our analyses. Research from other settings has shown considerable health harms observed well below these concentrations, and the high minimum concentrations of PM_2·5_ in our study presents a challenge in understanding these risks locally.^30,31^ In the absence of such local data, policy makers must rely on evidence from other settings in defining appropriate health-based thresholds.

The results of our study have direct relevance to policy in several ways. First, India is currently conducting its decadal process of reviewing its national ambient air quality standards (NAAQS). The NAAQS are substantially more relaxed than the WHO guidelines for acceptable exposure for all pollutants (eg 60 μg/m^3^ *vs* 15 μg/m^3^ for 24 h PM_2·5_ exposure). This study could serve as a strong addition to the growing local evidence base that the review could include in developing new standards for India. Second, the effect of PM_2·5_ at lower concentrations and the associated steep risk gradient means ambient PM_2·5_ must be reduced substantially from current concentrations to garner concomitant health benefits. Although India launched the National Clean Air Program in 2019, its target of reducing air pollution by 25–30% from 2017 concentrations will fall short in protecting health and preventing possible deaths from exposure to poor air quality. Furthermore, several cities have or are currently formulating Graded Response Action Plans to tackle high exposure events. These action plans kick in at high concentrations of air pollution (often above 150 μg/m^3^), which, based on our results, would only yield marginal benefits with respect to daily mortality, and negative health effects could continue to accrue even at lower pollution concentrations.^9,32,33^ Third, the estimates generated from our instrumental variable analysis have shown the substantial effect of local sources of air pollution, which are numerous in most Indian cities. Action plans to tackle air pollution must therefore direct as much attention to these dispersed sources of air pollution as they do to traditional point or line sources. Finally, the large fraction of deaths attributable to short-term PM_2·5_ exposures across all the cities we studied indicate that the emphasis on policy and action, which has gradually expanded to regions of India besides the Indo-Gangetic Plain, must intensify in coming years.

Short-term PM_2·5_ exposure increased the risk of daily mortality in multiple Indian cities of varying size and location. Our results generally show stronger associations than other studies, and highlighted the more pronounced associations for locally generated PM_2·5_. The plurality of study sites allowed us to extend analysis to lower ambient PM_2·5_ concentrations than previously studied in India, and the results revealed a steep increase in risk well below the current Indian PM_2·5_ standard. Daily deaths attributable to short-term PM_2·5_ exposure over the course of the study period amounted to approximately 30 000 (7·2%) deaths each year in the ten included cities. As efforts to develop and strengthen air pollution action plans at state, district, and city levels continue, the results of this study show the growing need to address dispersed local sources of air pollution in addition to traditional fixed and line sources. This work also provides important insights on harmful health outcomes even at lower pollution concentrations in India and reinforces the message that there is no safe level of exposure to air pollution, even in highly polluted regions.

## Supplementary Material

Supplement file

## Figures and Tables

**Table:** Attributable fraction (%) and deaths (N) to daily PM_2·5_ exposure with 95% CIs during the follow-up period, by city

	PM_2·5_, mg/m^3^ mean (SD)	Attributable fraction (95% CI)	Attributable deaths (95% CI)	Attributable deaths per year (95% CI)

Ahmedabad	37·9 (9·7)	5·6% (2·8–8·1)	28 680 (13 859–40 632)	2495 (1230–3588)
Bangalore	33·0 (6·5)	4·8% (2·2–7·2)	10 509 (5323–15 652)	2102 (969–3167)
Chennai	33·7 (9)	4·9% (2·2–7·3)	28 674 (12 883–43 266)	2870 (1329–4298)
Delhi	113·0 (64·5)	11·5% (5·2–16·4)	95 715 (45 449–13 5217)	11 964 (5399–16 983)
Hyderabad	38·9 (10·4)	5·6% (2·8–8·3)	5552 (2972–8274)	1597 (805–2363)
Kolkata	55·2 (35·3)	7·3% (4·0–10·5)	45 458 (26 227–63 911)	4678 (2573–6735)
Mumbai	41·7 (18·5)	5·6% (3·0–8·0)	30 544 (15 507–43 843)	5091 (2761–7340)
Pune	45·3 (22·6)	5·9% (3·3–8·6)	7169 (3866–10 328)	1367 (761–1999)
Shimla	28·4 (6·9)	3·7% (1·9–5·6)	281 (132–415)	59 (30–90)
Varanasi	82·1 (35·3)	10·2% (6·2–14·4)	8263 (4973–11 517)	831 (506–1178)
Total	53·6 (39·5)	7·2% (4·2–10·1)	26 0845 (151 397–367 490)	33 627 (19 443–47 426)

## Data Availability

All the data in this study are routinely collected and contain no information about specific people. Our data are available upon request to the corresponding author, subject to the agreement of the CHAIR-India steering group.

## Research in context

### Evidence before this study

We carried out two PubMed searches without language restrictions from database inception to March 14, 2024. We used the search terms “air pollution”, “particulate matter”, “short-term”, and “mortality” and then added “India”. Our search identified that numerous studies globally have found effects of short-term ambient air pollution on daily mortality. However, we observed no multi-city studies conducted in India and no global multi-city, multi-country analyses that featured Indian cities. Further, no study has applied causal inference methods to capture the role of locally generated pollutants in a low-income and-middle income setting. In addition, previous studies from India have not investigated exposure–response curves across such broad range of exposures, including evaluating the effect of air pollution at lower thresholds, even below the Indian recommended air quality guidelines. Finally, policy makers are reluctant to set standards based solely on studies conducted in other continents.

### Added value of this study

We observed an important effect of short-term exposure to PM_2·5_ on daily mortality in the first multi-city study including data from some of the largest and most polluted cities in India. These associations were observed to be stronger when using causal modelling methods accounting for locally generated pollutants. We were able to generate an integrated exposure–response curve for India that indicates increased risk of mortality even at lower concentrations of PM_2·5_ exposure. We did not observe any evidence of a safe threshold nor lower incremental effects on mortality at lower concentrations of PM_2·5_.

### Implications of all the available evidence

This study adds to the vast body of research globally showing increased effects of PM_2·5_ on daily mortality and provides strong evidence for such an association in India. Accounting for locally generated air pollutants by using causal methods might indicate a previous underestimation of the effect of air pollution. In line with previous studies regarding the exposure–response curve, no safe threshold for air pollution exposure exists. Our findings support the current evidence that approximately 7·2% of all deaths in India are attributable to daily PM_2·5_ exposure.

## References

[1] Di Q, Dai L, Wang Y, Association of short-term exposure to air pollution with mortality in older adults. JAMA 2017; 318: 2446–56. 29279932 10.1001/jama.2017.17923 PMC5783186

[2] Dehbi H-M, Blangiardo M, Gulliver J, Air pollution and cardiovascular mortality with over 25 years follow-up: a combined analysis of two British cohorts. Environ Int 2017; 99: 275–81. 27939045 10.1016/j.envint.2016.12.004 PMC5292102

[3] Mokoena KK, Ethan CJ, Yu Y, Shale K, Liu F. Ambient air pollution and respiratory mortality in Xi’an, China: a time-series analysis. Respir Res 2019; 20: 139. 31277656 10.1186/s12931-019-1117-8 PMC6612149

[4] Hystad P, Larkin A, Rangarajan S, Associations of outdoor fine particulate air pollution and cardiovascular disease in 157 436 individuals from 21 high-income, middle-income, and low-income countries (PURE): a prospective cohort study. Lancet Planet Health 2020; 4: e235–45. 32559440 10.1016/S2542-5196(20)30103-0 PMC7457447

[5] Lin C-K, Chang Y-T, Lee F-S, Chen S-T, Christiani D. Association between exposure to ambient particulate matters and risks of autism spectrum disorder in children: a systematic review and exposure-response meta-analysis. Environ Res Lett 2021; 16: 063003.

[6] Goyal N, Canning D. The association of in-utero exposure to ambient fine particulate air pollution with low birth weight in India. Environ Res Lett 2021; 16: 054034.

[7] Dey S, Purohit B, Balyan P, A satellite-based high-resolution (1-km) ambient PM_2·5_ database for India over two decades (2000–2019): applications for air quality management. Remote Sens (Basel) 2020; 12: 3872.

[8] Mandal S, Rajiva A, Kloog I, Nationwide estimation of daily ambient PM_2·5_ from 2008 to 2020 at 1km^2^ in India using an ensemble approach. PNAS Nexus 2024; 3: 088. 10.1093/pnasnexus/pgae088 PMC10919890 38456174

[9] Guttikunda SK, Dammalapati SK, Pradhan G, Krishna B, Jethva HT, Jawahar P. What is polluting Delhi’s air? A review from 1990 to 2022. Sustainability (Basel) 2023; 15: 4209.

[10] Gandhiok J. PM_2·5_ level improves in Delhi but still twice national average. 2023. https://www.hindustantimes.com/cities/delhi-news/pm25-level-improves-in-delhi-but-still-twice-national-average-101672598174528.html (accessed March 31, 2023).

[11] Krishna B, Mandal S, Madhipatla K, Reddy KS, Prabhakaran D, Schwartz JD. Daily nonaccidental mortality associated with short-term PM_2·5_ exposures in Delhi, India. Environ Epidemiol 2021; 5: e167. 34414349 10.1097/EE9.0000000000000167 PMC8367036

[12] Singh N, Mhawish A, Ghosh S, Banerjee T, Mall RK. Attributing mortality from temperature extremes: a time series analysis in Varanasi, India. Sci Total Environ 2019; 665: 453–64. 30772576 10.1016/j.scitotenv.2019.02.074

[13] deSouza PN, Dey S, Mwenda KM, Kim R, Subramanian SV, Kinney PL. Robust relationship between ambient air pollution and infant mortality in India. Sci Total Environ 2022; 815: 152755. 10.1016/j.scitotenv.2021.152755 34999065

[14] Pandey A, Brauer M, Cropper ML, Health and economic impact of air pollution in the states of India: the Global Burden of Disease Study 2019. Lancet Planet Health 2021; 5: e25–38. 33357500 10.1016/S2542-5196(20)30298-9 PMC7805008

[15] Liu C, Chen R, Sera F, Ambient particulate air pollution and daily mortality in 652 cities. N Engl J Med 2019; 381: 705–15. 31433918 10.1056/NEJMoa1817364 PMC7891185

[16] Chen R, Yin P, Meng X, Fine particulate air pollution and daily mortality. A nationwide analysis in 272 Chinese cities. Am J Respir Crit Care Med 2017; 196: 73–81. 28248546 10.1164/rccm.201609-1862OC

[17] Romieu I, Gouveia N, Cifuentes LA, Multicity study of air pollution and mortality in Latin America (the ESCALA study). Res Rep Health Eff Inst 2012; Oct: 5–86. 23311234

[18] Samoli E, Analitis A, Touloumi G, Estimating the exposure-response relationships between particulate matter and mortality within the APHEA multicity project. Environ Health Perspect 2005; 113: 88–95. 15626653 10.1289/ehp.7387 PMC1253715

[19] Dai L, Zanobetti A, Koutrakis P, Schwartz JD. Associations of fine particulate matter species with mortality in the United States: a multicity time-series analysis. Environ Health Perspect 2014; 122: 837–42. 24800826 10.1289/ehp.1307568 PMC4123030

[20] Singh N, Mhawish A, Ghosh S, Banerjee T, Mall RK. Attributing mortality from temperature extremes: a time series analysis in Varanasi, India. Sci Total Environ 2019; 665: 453–64. 30772576 10.1016/j.scitotenv.2019.02.074

[21] Schwartz J, Fong K, Zanobetti A. A national multicity analysis of the causal effect of local pollution, NO_2_, and PM_2·5_ on mortality. Environ Health Perspect 2018; 126: 087004. 10.1289/EHP2732 PMC6375387 30235421

[22] Schwartz J, Bind M-A, Koutrakis P. Estimating causal effects of local air pollution on daily deaths: effect of low levels. Environ Health Perspect 2017; 125: 23–29. 27203595 10.1289/EHP232 PMC5226700

[23] Registrar General & Census Commissioner of India. Census of India 2011. 2011. http://www.censusindia.gov.in/ (accessed March 18, 2024).

[24] Gasparrini A, Armstrong B, Kenward MG. Multivariate meta-analysis for non-linear and other multi-parameter associations. Stat Med 2012; 31: 3821–39. 22807043 10.1002/sim.5471 PMC3546395

[25] Gasparrini A, Leone M. Attributable risk from distributed lag models. BMC Med Res Methodol 2014; 14: 55. 24758509 10.1186/1471-2288-14-55 PMC4021419

[26] Joshi P, Dey S, Ghosh S, Jain S, Sharma SK. Association between acute exposure to PM_2·5_ chemical species and mortality in megacity Delhi, India. Environ Sci Technol 2022; 56: 7275–87. 35467339 10.1021/acs.est.1c06864

[27] Castro A, Künzli N, de Hoogh K, Mortality attributable to ambient fine particulate matter and nitrogen dioxide in Switzerland in 2019: use of two-pollutant effect estimates. Environ Res 2023; 231: 116029. 10.1016/j.envres.2023.116029 37149029

[28] Registrar General of India. Vital statistics of india based on the Civil Registration System 2020. 2020. https://crsorgi.gov.in/web/uploads/download/CRS_report_2020.pdf (accessed March 18, 2024).

[29] Saikia N, Kumar K, Das B. Death registration coverage 2019–2021, India. Bull World Health Organ 2023; 101: 102–10. 36733620 10.2471/BLT.22.288889 PMC9874366

[30] Brauer M, Brook JR, Christidis T, Mortality-Air Pollution Associations in Low Exposure Environments (MAPLE): phase 2. Res Rep Health Eff Inst 2022; 2022: 1–91. PMC9556709 36224709

[31] Awad YA, Di Q, Wang Y, Change in PM_2·5_ exposure and mortality among Medicare recipients: combining a semi-randomized approach and inverse probability weights in a low exposure population. Environ Epidemiol 2019; 3: e054. 31538135 10.1097/EE9.0000000000000054 PMC6693932

[32] Krishna B, Dey S. Making health the focus of air pollution policy. 2022. https://www.thehindu.com/opinion/op-ed/making-health-the-focus-of-air-pollution-policy/article66140242.ece (accessed Feb 27, 2023).

[33] Krishna B, Ganesan K, Prabhakaran P, Dey S. The bad science choking India. 2022. https://www.theindiaforum.in/article/bad-science-choking-india (accessed March 9, 2022).

