# Supplementary material for: Ambient air pollution and daily mortality in ten cities of India: a causal modelling study
Source: Lancet Planet Health. Author manuscript; Available in PMC 2025 Jan 29. (PMC11774940; doi:10.1016/S2542-5196(24)00114-1)
Supplement: Supplement file [file NIHMS2045244-supplement-Supplement_file.pdf]

THE LANCET
Planetary Health

Supplementary appendix

This appendix formed part of the original submission and has been peer reviewed. We post it as supplied by the authors.

Supplement to: de Bont J, Krishna B, Stafoggia M, et al. Ambient air pollution and daily mortality in ten cities of India: a causal modelling study. *Lancet Planet Health* 2024; **8:** e433–40.

Ambient air pollution and daily mortality in 10 cities of India: a causal modelling approach: supplementary material

Table of Contents

Table S1. Description of the 10 Indian cities included in the analyses. **2**

Table S2: Attributable fraction (%) and deaths (N) to daily $PM_{2.5}$ exposure with 95% confidence intervals (CI) during the follow-up period (reference level is the Indian guidelines (<60 μg/m^3). **3**

Figure S1: Number of days with levels at each threshold of $PM_{2.5}$ **4**

Figure S2. Sensitivity analyses: lag patterns of the association between $PM_{2.5}$ and daily mortality **5**

Figure S3. Sensitivity analyses: different adjustments evaluating the association between air pollution and daily mortality. **6**

Figure S4. Sensitivity analyses: adjusting for relative humidity for those cities where data was available. **7**

Figure S5. Sensitivity analyses: Cumulative exposure-response relationship and attributable fraction and deaths to daily $PM_{2.5}$ exposure using different knot points: A) equidistant knot points (25th, 50th, 75th percentile), and B) at specific percentiles (10th, 50th and 90th). **8**

Table S1. Description of the 10 Indian cities included in the analyses.

City	Population N (2011)	Area (km^2)	Time period	Mortality [N° (daily mean, SD)]	Mortality rate [Deaths per 100,000 population (SD)]
Ahmedabad	6,550,084	505	01/01/2008-31/06/2019	510217 (122, 24)	74·8 (3·3)
Bangalore	7,552,321	741	01/01/2008-31/12/2012	220521 (121, 17)	53·8 (2·4)
Chennai	7,139,630	426	01/01/2010-31/12/2019	592336 (164, 23)	115·6 (4·4)
Delhi	16,349,831	1397	01/01/2011-31/12/2018	830280 (284, 44)	85·3 (6·8)
Hyderabad	7,677,018	650	01/01/2008-30/06/2011	99006 (78, 13)	45·1 (4·1)
Kolkata	4,496,694	206	13/01/2010-31/12/2019	625213 (172, 32)	125·6 (4·7)
Mumbai	12,432,830	440	01/01/2009-30/11/2015	548592 (251, 28)	70·5 (5·2)
Pune	6,451,618	485	01/01/2008-31/12/2012	121961 (68, 11)	82·8 (2·6)
Shimla	171,817	35	01/01/2008-31/08/2012	7623 (5, 2)	98·9 (9·1)
Varanasi	1,746,467	121	01/01/2008-30/11/2018*	81413 (223, 6)	66·5 (3·8)

* Mortality data in Varanasi during 2017 was excluded because of low quality.

Table S2: Attributable fraction (%) and deaths (N) to daily $PM_{2 \cdot 5}$ exposure with 95% confidence intervals (CI) during the follow-up period (reference level is the Indian guidelines (<60 μg/m^3).

**City**	**Attributable fraction in % (95%CI)**	**Attributable deaths (95%CI)**	**Attributable deaths per year (95%CI)**
Ahmedabad	2·2 (0·9; 3·4)	118 (49; 183)	965 (388; 1488)
Bangalore	1·0 (0·6; 1·4)	2 (1; 4)	424 (244; 610)
Chennai	0·6 (0·3; 0·8)	66 (39; 95)	331 (188; 470)
Delhi	4·6 (-3·6; 10·1)	31118 (-19136; 69244)	4774 (-3700; 10460)
Hyderabad	0·5 (0·3; 0·8)	4 (2; 5)	154 (89; 222)
Kolkata	3·3 (1·9; 4·8)	8644 (5003; 12156)	2107 (1204; 3046)
Mumbai	0·7 (0·4; 1·0)	931 (517; 1339)	650 (374; 930)
Pune	1·2 (0·7; 1·7)	509 (295; 719)	280 (166; 404)
Shimla*	-	-	-
Varanasi	3·3 (1·6; 4·9)	1939 (932; 2925)	268 (129; 404)
**Total**	3·6 (-1·1; 7·2)	43331 (-15073; 82568)	17060 (-5124; 33656)

*No attributable fraction could be calculated for Shimla as no days were above the reference level in the Indian guidelines (<60 μg/m^3).

Figure S1: Number of days with levels at each threshold of $PM_{2 \cdot 5}$.

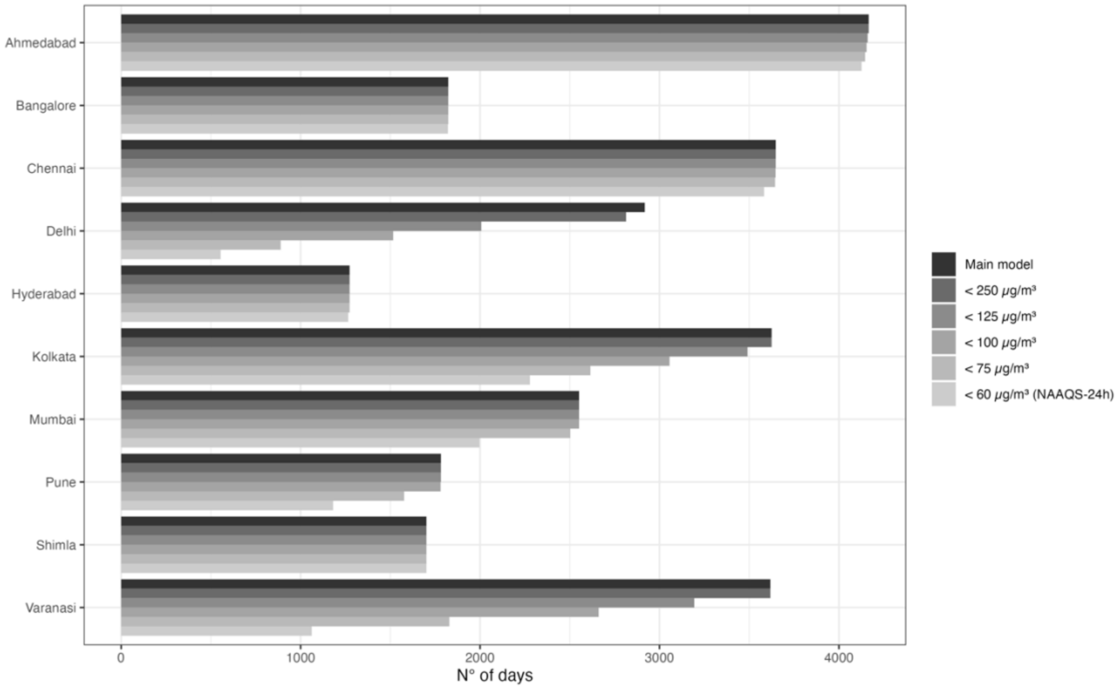

Figure S2. Sensitivity analyses: lag patterns of the association between $PM_{2.5}$ and daily mortality

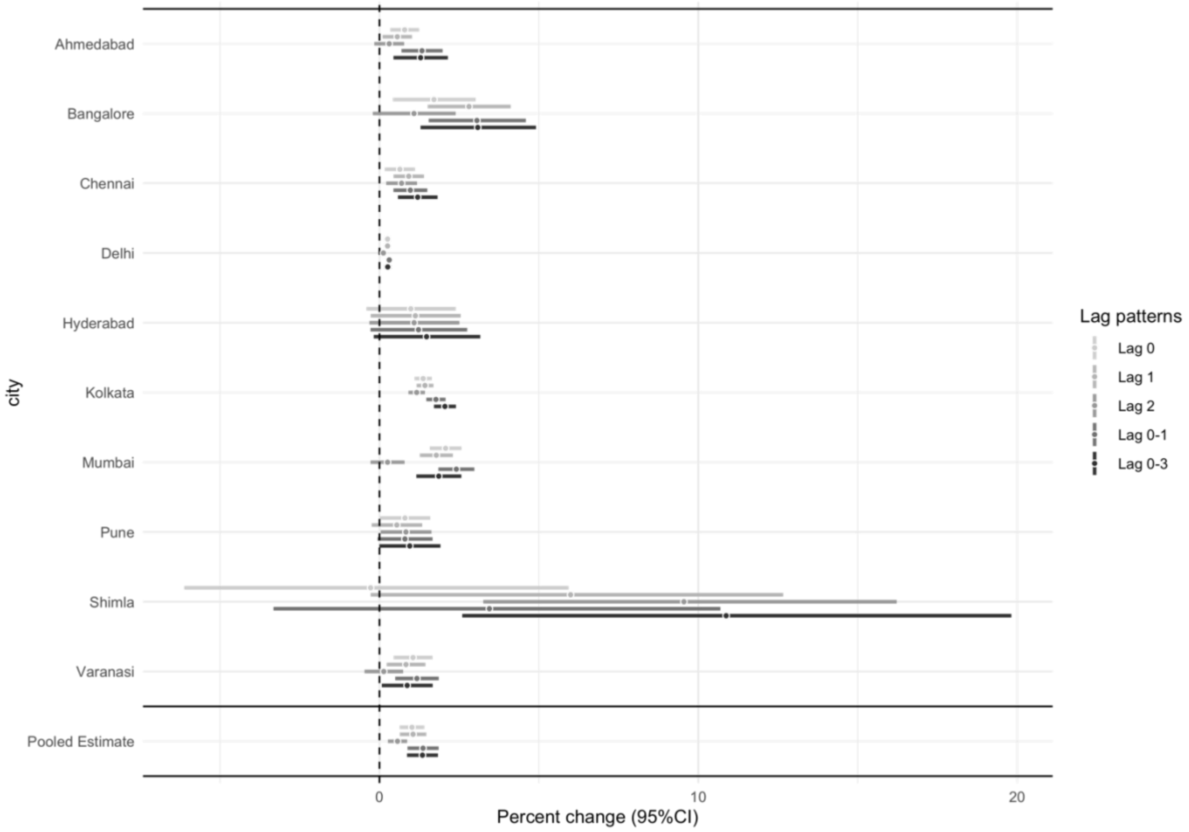

Estimates are given as percentage change in mortality and 95% confidence interval per 10 μg/m^3 increase in $PM_{2.5}$ (lag 01). We checked single lags of 0, 1 and 2 days, 2-day moving average (Lag 0-1, main model) and 4-day moving average (Lag 0-3).

Figure S3. Sensitivity analyses: different adjustments evaluating the association between air pollution and daily mortality.

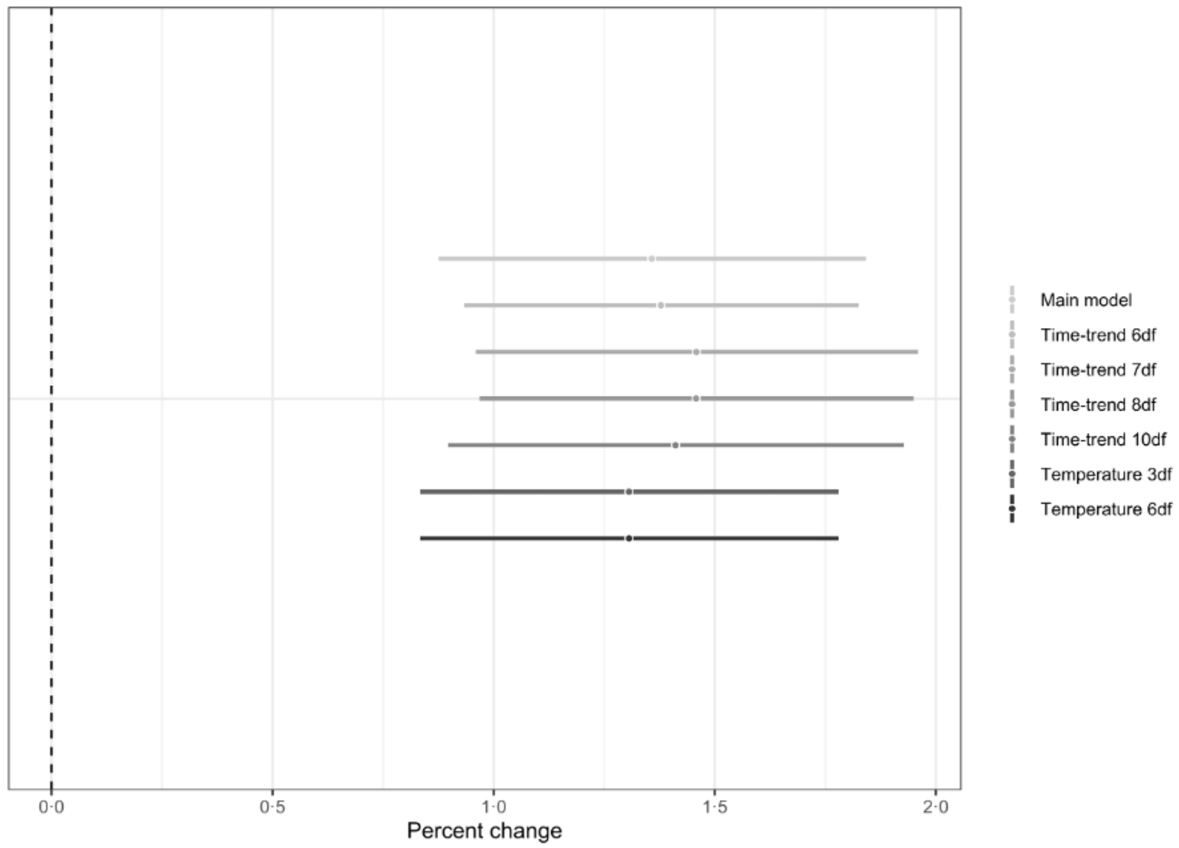

Estimates are given as percentage change in mortality and 95% confidence interval per 10 μg/m^3 increase in $PM_{2·5}$ (lag 01). The main model evaluated time trends with 9 df. We evaluated different degrees of freedoms adjusting for time trends (6-10 df/year). We additionally evaluated alternative number of *df* (3 and 6) in natural spline function for temperature (lag04).

Figure S4. Sensitivity analyses: adjusting for relative humidity for those cities where data was available.

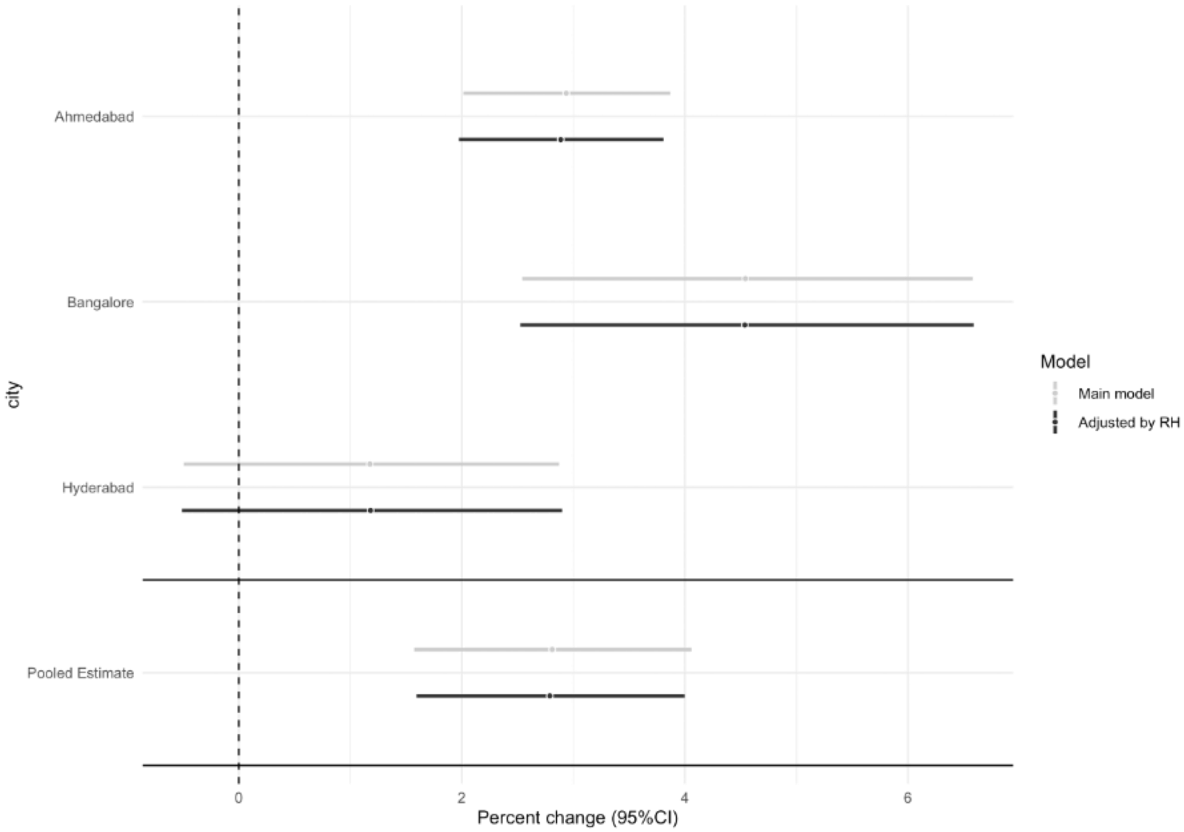

Estimates are given as percentage change in mortality and 95% confidence interval per 10 µg/m^3 increase in $PM_{2\cdot5}$ (lag 01).

Figure S5. Sensitivity analyses: Cumulative exposure-response relationship and attributable fraction and deaths to daily $PM_{2\cdot5}$ exposure using different knot points: A) equidistant knot points (25th, 50th, 75th percentile), and B) at specific percentiles (10th, 50th and 90th).

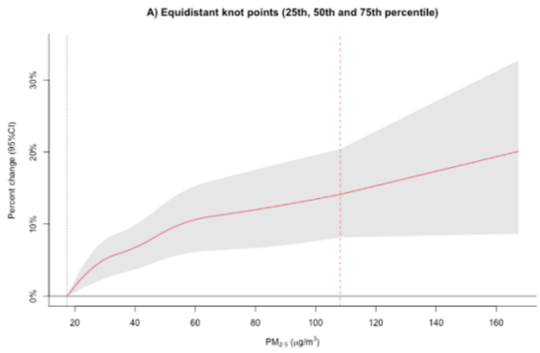

City	Attributable fraction in % (95%CI)	Attributable deaths (95%CI)
Ahmedabad	6·0 (3·4; 8·7)	30674 (17412; 43574)
Bangalore	5·1 (2·9; 7·3)	11306 (5904; 16750)
Chennai	5·3 (2·8; 7·8)	31136 (16815; 44925)
Delhi	13·3 (6·1; 18·5)	109907 (46715; 152912)
Hyderabad	6·1 (3·5; 8·7)	6015 (3472; 8421)
Kolkata	7·6 (4·8; 10·7)	47649 (28882; 65153)
Mumbai	6·1 (3·6; 8·5)	33408 (19792; 47036)
Pune	6·3 (3·6; 8·8)	7663 (4527; 10624)
Shimla	4·0 (2·2; 5·8)	302 (154; 454)
Varanasi	10·8 (6·7; 14·6)	8752 (5639; 11871)
**Total**	8·0 (4·7; 11·0)	292665 (176063; 401276)

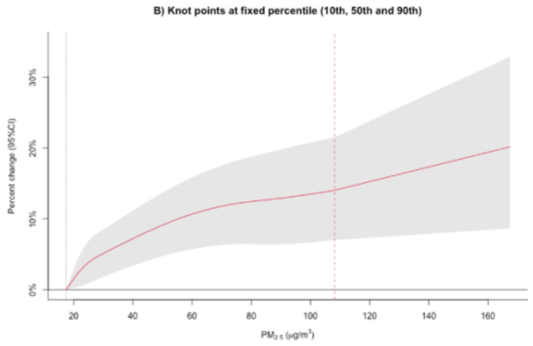

City	Attributable fraction in % (95%CI)	Attributable deaths (95%CI)
Ahmedabad	6·2 (2·8; 9·5)	31769 (13969; 46952)
Bangalore	5·4 (2·4; 8·1)	11802 (4842; 18541)
Chennai	5·4 (2·6; 8·5)	32195 (13305; 49809)
Delhi	13·6 (5·7; 18·8)	112929 (41779; 156307)
Hyderabad	6·3 (2·9; 9·4)	6225 (3240; 9319)
Kolkata	7·8 (4·3; 11·2)	48458 (27244; 71026)
Mumbai	6·3 (3·3; 9·5)	34407 (17784; 50589)
Pune	6·5 (3·5; 9·4)	7899 (4072; 11612)
Shimla	4·1 (1·7; 6·6)	313 (112; 500)
Varanasi	10·9 (6·1; 15·2)	8868 (4740; 12441)
**Total**	8·2 (4·5; 11·6)	299506 (157170; 427456)

The figures represent the relative risk of air pollution against the minimum air pollution level (grey line) at which an effect was observed in our study (17.1 $\mu g/m^3$). Ideally, this would be assessed at the WHO 24-hour ambient $PM_{2\cdot5}$ guideline of 15 $\mu g/m^3$, but this was not feasible as there were very few days where such levels were observed in our data set. The red vertical line was the 99th percentile.
